# (4,4′-Dimethyl-2,2′-bipyridine-κ^2^ *N*,*N*′)diiodidomercury(II)

**DOI:** 10.1107/S1600536808028791

**Published:** 2008-09-13

**Authors:** Mohammad Yousefi, Nasim Tadayon Pour, Vahid Amani, Hamid Reza Khavasi

**Affiliations:** a Islamic Azad University, Shahr-e-Rey Branch, Tehran, Iran; b Department of Chemistry, Shahid Beheshti University, Tehran 1983963113, Iran

## Abstract

In the mol­ecule of the title compound, [HgI_2_(C_12_H_12_N_2_)], the Hg^II^ atom is four-coordinated in a distorted tetra­hedral configuration by two N atoms from the 4,4′-dimethyl-2,2′-bipyridine ligand and by two I atoms. There is a π–π contact between the pyridine rings [centroid–centroid distance = 3.775 (6) Å].

## Related literature

For related literature, see: Khalighi *et al.* (2008 ▶); Ahmadi *et al.* (2008 ▶); Khavasi *et al.* (2008 ▶); Freire *et al.* (1999 ▶); Chen *et al.* (2006 ▶); Htoon & Ladd (1976 ▶).
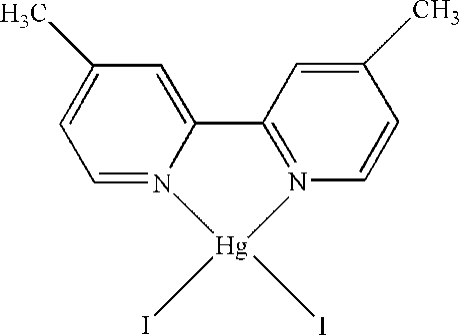


## Experimental

### 

#### Crystal data

[HgI_2_(C_12_H_12_N_2_)]
*M* *_r_* = 638.63
Triclinic, 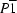

*a* = 8.4214 (9) Å
*b* = 9.8391 (10) Å
*c* = 10.2983 (10) Å
α = 69.383 (8)°
β = 88.448 (8)°
γ = 74.670 (8)°
*V* = 768.18 (14) Å^3^
*Z* = 2
Mo *K*α radiation
μ = 14.02 mm^−1^
*T* = 298 (2) K
0.38 × 0.25 × 0.12 mm

#### Data collection

Bruker SMART CCD area-detector diffractometer
Absorption correction: multi-scan (*SADABS*; Sheldrick, 1998 ▶) *T*_min_ = 0.022, *T*_max_ = 0.183
8874 measured reflections
4123 independent reflections
3467 reflections with *I* > 2σ(*I*)
*R*_int_ = 0.091

#### Refinement

*R*[*F* ^2^ > 2σ(*F* ^2^)] = 0.048
*wR*(*F* ^2^) = 0.159
*S* = 0.95
4123 reflections
155 parameters
H-atom parameters constrained
Δρ_max_ = 1.95 e Å^−3^
Δρ_min_ = −1.38 e Å^−3^

### 

Data collection: *SMART* (Bruker, 1998 ▶); cell refinement: *SAINT* (Bruker, 1998 ▶); data reduction: *SAINT*; program(s) used to solve structure: *SHELXTL* (Sheldrick, 2008 ▶); program(s) used to refine structure: *SHELXTL*; molecular graphics: *ORTEP-3 for Windows* (Farrugia, 1997 ▶); software used to prepare material for publication: *WinGX* (Farrugia, 1999 ▶).

## Supplementary Material

Crystal structure: contains datablocks I, global. DOI: 10.1107/S1600536808028791/hk2525sup1.cif

Structure factors: contains datablocks I. DOI: 10.1107/S1600536808028791/hk2525Isup2.hkl

Additional supplementary materials:  crystallographic information; 3D view; checkCIF report

## Figures and Tables

**Table:** 

Hg1—I1	2.6671 (9)
Hg1—I2	2.6885 (8)
N1—Hg1	2.442 (8)
N2—Hg1	2.402 (10)

**Table:** 

I1—Hg1—I2	132.56 (3)
N1—Hg1—I1	111.9 (2)
N1—Hg1—I2	92.87 (19)
N2—Hg1—N1	67.3 (3)
N2—Hg1—I1	104.9 (2)
N2—Hg1—I2	122.2 (2)

**Fig. 1.**
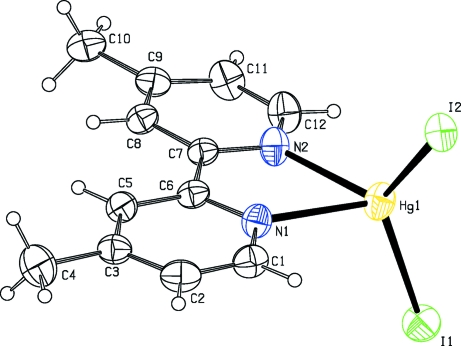
The molecular structure of the title molecule, with the atom-numbering scheme. Displacement ellipsoids are drawn at the 40% probability level.

**Fig. 2.**
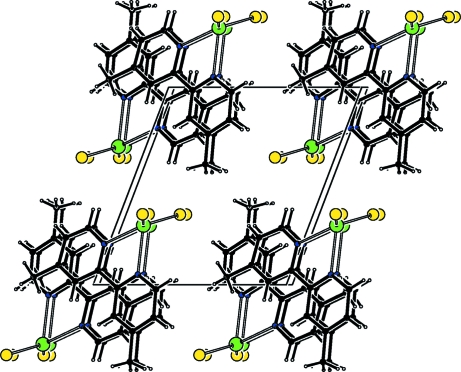
A packing diagram of the title compound.

## supplementary crystallographic information

### Comment

Recently, we reported the syntheses and crystal structures of [Zn(5,5'-dmbpy)Cl_2_], (II), (Khalighi *et al.*, 2008), [Cd(5,5'-dmbpy)(µ-Cl)_2_]_n_, (III), (Ahmadi *et al.*, 2008) and {[HgCl(dm4bt)]_2_(µ-Cl)_2_}, (IV), (Khavasi *et al.*, 2008) [where 5,5'-dmbpy is 5,5'-dimethyl-2,2'-bipyridine and dm4bt is 2,2'-dimethyl-4,4' -bithiazole]. There are several Hg^II^ complexes, with formula, [HgI_2_(N-N)], such as [HgI_2_(bipy)], (V), [HgI_2_(phen)], (VI), [HgI_2_(2,9-dmphen)], (VII), (Freire *et al.*, 1999), [HgI_2_(bipy)][HgI_2_], (VIII), (Chen *et al.*, 2006) and [HgI_2_(TMDA)], (IX), (Htoon & Ladd, 1976) [where bipy is 2,2'-bipyridine, phen is 1,10-phenanthroline, dmphen is 2,9-dimethyl-1,10-phenanthroline and TMDA is tetramethylethylenediamine] have been synthesized and characterized by single-crystal X-ray diffraction methods. We report herein the synthesis and crystal structure of the title compound, (I).

In the title compound, (I), (Fig. 1), the Hg^II^ atom is four-coordinated in a distorted tetrahedral configuration by two N atoms from 4,4'-dimethyl-2,2'-bipyridine and two I atoms. The Hg—I and Hg—N bond lengths and angles (Table 1) are within normal ranges, as in (V) and (VI).

In the crystal structure, the π–π contact (Fig. 2) between the pyridine rings, Cg2···Cg3^i^ [symmetry code: (i) 1 - x, -y, -z, where Cg2 and Cg3 are the centroids of the rings (N1/C1–C3/C5/C6) and (N2/C7–C9/C11/C12), respectively] may stabilize the structure, with centroid–centroid distance of 3.775 (6) Å.

### Experimental

For the preparation of the title compound, (I), a solution of 4,4'-dimethyl-2,2'-bipyridine (0.25 g, 1.33 mmol) in methanol (20 ml) was added to a solution of HgI_2_ (0.61 g, 1.33 mmol) in acetonitrile (50 ml) and the resulting colorless solution was stirred for 20 min at room temperature, and then it was left to evaporate slowly. After one week, colorless block crystals of (I) were isolated (yield; 0.61 g, 71.8%).

### Refinement

H atoms were positioned geometrically, with C—H = 0.93 and 0.96 Å for aromatic and methyl H, respectively, and constrained to ride on their parent atoms with U_iso_(H) = 1.2U_eq_(C).

### Crystal data

**Table:**

[HgI_2_(C_12_H_12_N_2_)]	*Z* = 2
*M**_r_* = 638.63	*F*(000) = 568
Triclinic, *P*1	*D*_x_ = 2.761 Mg m^−^^3^
Hall symbol: -P 1	Mo *K*α radiation, λ = 0.71073 Å
*a* = 8.4214 (9) Å	Cell parameters from 2145 reflections
*b* = 9.8391 (10) Å	θ = 2.1–29.2°
*c* = 10.2983 (10) Å	µ = 14.02 mm^−^^1^
α = 69.383 (8)°	*T* = 298 K
β = 88.448 (8)°	Block, colourless
γ = 74.670 (8)°	0.38 × 0.25 × 0.12 mm
*V* = 768.18 (14) Å^3^	

### Data collection

**Table:**

Bruker SMART CCD area-detector diffractometer	4123 independent reflections
Radiation source: fine-focus sealed tube	3467 reflections with *I* > 2σ(*I*)
graphite	*R*_int_ = 0.091
φ and ω scans	θ_max_ = 29.2°, θ_min_ = 2.1°
Absorption correction: multi-scan (SADABS; Sheldrick, 1998)	*h* = −11→11
*T*_min_ = 0.022, *T*_max_ = 0.183	*k* = −13→13
8874 measured reflections	*l* = −14→14

### Refinement

**Table:**

Refinement on *F*^2^	Secondary atom site location: difference Fourier map
Least-squares matrix: full	Hydrogen site location: inferred from neighbouring sites
*R*[*F*^2^ > 2σ(*F*^2^)] = 0.048	H-atom parameters constrained
*wR*(*F*^2^) = 0.159	*w* = 1/[σ^2^(*F*_o_^2^) + (0.108*P*)^2^ + 1.3499*P*] where *P* = (*F*_o_^2^ + 2*F*_c_^2^)/3
*S* = 0.95	(Δ/σ)_max_ = 0.002
4123 reflections	Δρ_max_ = 1.95 e Å^−^^3^
155 parameters	Δρ_min_ = −1.38 e Å^−^^3^
0 restraints	Extinction correction: SHELXTL (Sheldrick, 1998), Fc^*^=kFc[1+0.001xFc^2^λ^3^/sin(2θ)]^-1/4^
Primary atom site location: structure-invariant direct methods	Extinction coefficient: 0.0071 (13)

### Special details

**Table:**

Geometry. All e.s.d.'s (except the e.s.d. in the dihedral angle between two l.s. planes) are estimated using the full covariance matrix. The cell e.s.d.'s are taken into account individually in the estimation of e.s.d.'s in distances, angles and torsion angles; correlations between e.s.d.'s in cell parameters are only used when they are defined by crystal symmetry. An approximate (isotropic) treatment of cell e.s.d.'s is used for estimating e.s.d.'s involving l.s. planes.
Refinement. Refinement of *F*^2^ against ALL reflections. The weighted *R*-factor *wR* and goodness of fit *S* are based on *F*^2^, conventional *R*-factors *R* are based on *F*, with *F* set to zero for negative *F*^2^. The threshold expression of *F*^2^ > σ(*F*^2^) is used only for calculating *R*-factors(gt) *etc*. and is not relevant to the choice of reflections for refinement. *R*-factors based on *F*^2^ are statistically about twice as large as those based on *F*, and *R*- factors based on ALL data will be even larger.

### Fractional atomic coordinates and isotropic or equivalent isotropic displacement parameters (Å^2^)

**Table:**

	*x*	*y*	*z*	*U*_iso_*/*U*_eq_	
Hg1	0.31760 (5)	0.15247 (5)	0.30109 (4)	0.05970 (19)	
I1	0.03155 (9)	0.31134 (10)	0.35551 (9)	0.0704 (2)	
I2	0.63357 (8)	0.13618 (9)	0.35681 (7)	0.0594 (2)	
N1	0.3481 (10)	0.2223 (10)	0.0514 (8)	0.0520 (16)	
N2	0.2438 (12)	−0.0160 (12)	0.2073 (9)	0.0595 (19)	
C1	0.3943 (12)	0.3461 (12)	−0.0232 (12)	0.058 (2)	
H1	0.4209	0.4042	0.0227	0.069*	
C2	0.4037 (14)	0.3905 (12)	−0.1657 (13)	0.064 (3)	
H2	0.4374	0.4763	−0.2141	0.077*	
C3	0.3630 (12)	0.3066 (10)	−0.2355 (10)	0.053 (2)	
C4	0.368 (2)	0.3537 (19)	−0.3901 (13)	0.080 (4)	
H4A	0.2607	0.3701	−0.4313	0.096*	
H4B	0.4460	0.2758	−0.4127	0.096*	
H4C	0.4015	0.4454	−0.4256	0.096*	
C5	0.3166 (12)	0.1780 (10)	−0.1574 (9)	0.0512 (18)	
H5	0.2901	0.1178	−0.2009	0.061*	
C6	0.3098 (10)	0.1393 (10)	−0.0149 (9)	0.0461 (16)	
C7	0.2537 (10)	0.0050 (9)	0.0721 (9)	0.0438 (15)	
C8	0.2150 (11)	−0.0932 (10)	0.0177 (10)	0.0488 (17)	
H8	0.2260	−0.0777	−0.0762	0.059*	
C9	0.1602 (11)	−0.2137 (10)	0.1003 (11)	0.0525 (19)	
C10	0.1200 (14)	−0.3201 (12)	0.0393 (13)	0.065 (2)	
H10A	0.2170	−0.3670	0.0034	0.079*	
H10B	0.0348	−0.2648	−0.0346	0.079*	
H10C	0.0825	−0.3962	0.1103	0.079*	
C11	0.1411 (15)	−0.2305 (14)	0.2394 (12)	0.065 (3)	
H11	0.1004	−0.3078	0.2981	0.078*	
C12	0.1831 (16)	−0.1319 (15)	0.2875 (11)	0.066 (3)	
H12	0.1698	−0.1438	0.3805	0.079*	

### Atomic displacement parameters (Å^2^)

**Table:**

	*U*^11^	*U*^22^	*U*^33^	*U*^12^	*U*^13^	*U*^23^
Hg1	0.0532 (2)	0.0783 (3)	0.0614 (3)	−0.02592 (18)	0.01400 (15)	−0.0362 (2)
I1	0.0537 (4)	0.0824 (5)	0.0729 (4)	−0.0159 (3)	0.0167 (3)	−0.0280 (4)
I2	0.0539 (3)	0.0771 (4)	0.0543 (3)	−0.0294 (3)	0.0096 (2)	−0.0241 (3)
N1	0.056 (4)	0.058 (4)	0.049 (3)	−0.022 (3)	0.010 (3)	−0.022 (3)
N2	0.063 (5)	0.077 (5)	0.047 (4)	−0.029 (4)	0.011 (3)	−0.026 (4)
C1	0.054 (5)	0.052 (5)	0.074 (6)	−0.023 (4)	0.005 (4)	−0.025 (4)
C2	0.061 (5)	0.047 (5)	0.079 (7)	−0.021 (4)	0.010 (5)	−0.013 (4)
C3	0.054 (5)	0.044 (4)	0.056 (5)	−0.013 (3)	0.006 (4)	−0.012 (3)
C4	0.089 (9)	0.089 (9)	0.056 (5)	−0.033 (7)	0.013 (5)	−0.013 (6)
C5	0.057 (5)	0.048 (4)	0.048 (4)	−0.015 (4)	0.009 (3)	−0.016 (3)
C6	0.041 (4)	0.044 (4)	0.052 (4)	−0.011 (3)	0.013 (3)	−0.017 (3)
C7	0.039 (3)	0.042 (3)	0.048 (4)	−0.010 (3)	0.008 (3)	−0.016 (3)
C8	0.047 (4)	0.048 (4)	0.053 (4)	−0.014 (3)	0.012 (3)	−0.020 (3)
C9	0.043 (4)	0.048 (4)	0.061 (5)	−0.012 (3)	0.004 (3)	−0.014 (3)
C10	0.067 (5)	0.049 (5)	0.078 (6)	−0.014 (4)	0.010 (5)	−0.021 (4)
C11	0.071 (6)	0.070 (6)	0.060 (5)	−0.040 (5)	0.007 (5)	−0.013 (5)
C12	0.075 (7)	0.079 (7)	0.050 (4)	−0.040 (5)	0.015 (4)	−0.017 (4)

### Geometric parameters (Å, °)

**Table:**

Hg1—I1	2.6671 (9)	C6—N1	1.334 (12)
Hg1—I2	2.6885 (8)	C6—C7	1.498 (12)
N1—Hg1	2.442 (8)	C7—N2	1.337 (12)
N2—Hg1	2.402 (10)	C7—C8	1.383 (13)
C1—N1	1.342 (13)	C8—C9	1.378 (13)
C1—C2	1.382 (17)	C8—H8	0.9300
C1—H1	0.9300	C9—C11	1.392 (16)
C2—C3	1.377 (17)	C9—C10	1.506 (16)
C2—H2	0.9300	C10—H10A	0.9600
C3—C5	1.389 (13)	C10—H10B	0.9600
C3—C4	1.497 (16)	C10—H10C	0.9600
C4—H4A	0.9600	C11—C12	1.357 (18)
C4—H4B	0.9600	C11—H11	0.9300
C4—H4C	0.9600	C12—N2	1.366 (15)
C5—C6	1.384 (12)	C12—H12	0.9300
C5—H5	0.9300		
			
I1—Hg1—I2	132.56 (3)	C6—C5—C3	120.2 (9)
N1—Hg1—I1	111.9 (2)	C6—C5—H5	119.7
N1—Hg1—I2	92.87 (19)	C3—C5—H5	120.2
N2—Hg1—N1	67.3 (3)	N1—C6—C5	121.6 (8)
N2—Hg1—I1	104.9 (2)	N1—C6—C7	116.6 (8)
N2—Hg1—I2	122.2 (2)	C5—C6—C7	121.7 (8)
C1—N1—Hg1	122.2 (7)	N2—C7—C8	121.2 (8)
C6—N1—Hg1	119.2 (6)	N2—C7—C6	116.1 (8)
C6—N1—C1	118.6 (8)	C8—C7—C6	122.6 (8)
C7—N2—Hg1	120.8 (7)	C9—C8—C7	121.0 (9)
C7—N2—C12	117.4 (9)	C9—C8—H8	119.4
C12—N2—Hg1	121.7 (7)	C7—C8—H8	119.6
N1—C1—C2	122.5 (10)	C8—C9—C11	117.7 (10)
N1—C1—H1	118.7	C8—C9—C10	120.2 (10)
C2—C1—H1	118.8	C11—C9—C10	122.1 (9)
C3—C2—C1	119.5 (10)	C9—C10—H10A	109.2
C3—C2—H2	120.1	C9—C10—H10B	109.6
C1—C2—H2	120.4	H10A—C10—H10B	109.5
C2—C3—C5	117.6 (10)	C9—C10—H10C	109.6
C2—C3—C4	120.8 (11)	H10A—C10—H10C	109.5
C5—C3—C4	121.6 (11)	H10B—C10—H10C	109.5
C3—C4—H4A	109.3	C12—C11—C9	118.7 (10)
C3—C4—H4B	109.5	C12—C11—H11	120.7
H4A—C4—H4B	109.5	C9—C11—H11	120.6
C3—C4—H4C	109.6	N2—C12—C11	123.8 (10)
H4A—C4—H4C	109.5	N2—C12—H12	118.1
H4B—C4—H4C	109.5	C11—C12—H12	118.2
			
C1—N1—Hg1—I1	79.7 (8)	C5—C6—N1—Hg1	176.7 (6)
C1—N1—Hg1—I2	−58.9 (7)	C7—C6—N1—Hg1	−1.4 (10)
C1—N1—Hg1—N2	177.2 (8)	C5—C6—N1—C1	−0.1 (13)
C6—N1—Hg1—I1	−96.9 (7)	C7—C6—N1—C1	−178.1 (8)
C6—N1—Hg1—I2	124.5 (7)	N1—C6—C7—N2	1.6 (11)
C6—N1—Hg1—N2	0.6 (7)	C5—C6—C7—N2	−176.5 (9)
C7—N2—Hg1—I1	108.1 (7)	N1—C6—C7—C8	−178.0 (8)
C7—N2—Hg1—I2	−78.2 (8)	C5—C6—C7—C8	4.0 (12)
C7—N2—Hg1—N1	0.2 (7)	C6—C7—N2—Hg1	−1.0 (10)
C12—N2—Hg1—I1	−68.7 (9)	C8—C7—N2—Hg1	178.6 (6)
C12—N2—Hg1—I2	104.9 (9)	C6—C7—N2—C12	176.0 (9)
C12—N2—Hg1—N1	−176.6 (10)	C8—C7—N2—C12	−4.5 (14)
C2—C1—N1—C6	−0.1 (15)	N2—C7—C8—C9	1.9 (13)
C2—C1—N1—Hg1	−176.7 (8)	C6—C7—C8—C9	−178.6 (8)
N1—C1—C2—C3	0.7 (16)	C7—C8—C9—C11	1.7 (13)
C1—C2—C3—C5	−1.2 (16)	C7—C8—C9—C10	−179.5 (8)
C1—C2—C3—C4	178.6 (11)	C8—C9—C11—C12	−2.5 (16)
C2—C3—C5—C6	1.1 (14)	C10—C9—C11—C12	178.7 (11)
C4—C3—C5—C6	−178.7 (10)	C9—C11—C12—N2	−0.1 (19)
C3—C5—C6—N1	−0.5 (14)	C11—C12—N2—Hg1	−179.4 (10)
C3—C5—C6—C7	177.4 (8)	C11—C12—N2—C7	3.6 (18)
